# 2-Methyl-3-(2-methyl­phen­yl)-7-nitro­quinazolin-4(3*H*)-one

**DOI:** 10.1107/S1600536812007350

**Published:** 2012-02-29

**Authors:** Adel S. El-Azab, Alaa A.-M. Abdel-Aziz, Seik Weng Ng, Edward R. T. Tiekink

**Affiliations:** a Department of Pharmaceutical Chemistry, College of Pharmacy, King Saud University, Riyadh 11451, Saudi Arabia; b Department of Organic Chemistry, Faculty of Pharmacy, Al-Azhar University, Cairo 11884, Egypt; c Department of Medicinal Chemistry, Faculty of Pharmacy, University of Mansoura, Mansoura 35516, Egypt; d Department of Chemistry, University of Malaya, 50603 Kuala Lumpur, Malaysia; e Chemistry Department, Faculty of Science, King Abdulaziz University, PO Box 80203 Jeddah, Saudi Arabia

## Abstract

In the title methaqua­lone analogue, C_16_H_13_N_3_O_3_, the 2-tolyl group is almost orthogonal [dihedral angle = 85.20 (5)°] to the fused ring system (r.m.s. deviation of fitted non-H atoms = 0.029 Å). In the crystal, twofold symmetry generates two-mol­ecule aggregates linked by C—H⋯O and π–π inter­actions [ring centroid–centroid distance = 3.4967 (6) Å].

## Related literature

For recent studies on synthesis, drug discovery and crystal structures of quinazoline-4(3*H*)-one derivatives, see: El-Azab *et al.* (2010 ▶, 2012 ▶). For the anti-convulsant activity of the title methaqua­lone analogue, see: El-Azab *et al.* (2011 ▶). For a related structure, see: Stephenson *et al.* (2011 ▶).
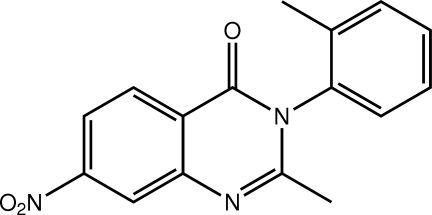


## Experimental

### 

#### Crystal data

C_16_H_13_N_3_O_3_
*M* *_r_* = 295.29
Monoclinic, 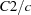

*a* = 14.4614 (5) Å
*b* = 16.5383 (4) Å
*c* = 12.9968 (4) Å
β = 119.072 (4)°
*V* = 2716.78 (14) Å^3^
*Z* = 8
Cu *K*α radiation
μ = 0.85 mm^−1^
*T* = 100 K
0.25 × 0.25 × 0.25 mm

#### Data collection

Agilent SuperNova Dual diffractometer with an Atlas detector
Absorption correction: multi-scan (*CrysAlis PRO*; Agilent, 2011 ▶) *T*_min_ = 0.967, *T*_max_ = 0.998
5450 measured reflections
2783 independent reflections
2570 reflections with *I* > 2σ(*I*)
*R*_int_ = 0.013

#### Refinement

*R*[*F* ^2^ > 2σ(*F* ^2^)] = 0.035
*wR*(*F* ^2^) = 0.099
*S* = 1.02
2783 reflections
201 parameters
H-atom parameters constrained
Δρ_max_ = 0.24 e Å^−3^
Δρ_min_ = −0.24 e Å^−3^

### 

Data collection: *CrysAlis PRO* (Agilent, 2011 ▶); cell refinement: *CrysAlis PRO*; data reduction: *CrysAlis PRO*; program(s) used to solve structure: *SHELXS97* (Sheldrick, 2008 ▶); program(s) used to refine structure: *SHELXL97* (Sheldrick, 2008 ▶); molecular graphics: *ORTEP-3* (Farrugia, 1997 ▶) and *DIAMOND* (Brandenburg, 2006 ▶); software used to prepare material for publication: *publCIF* (Westrip, 2010 ▶).

## Supplementary Material

Crystal structure: contains datablock(s) global, I. DOI: 10.1107/S1600536812007350/hb6642sup1.cif

Structure factors: contains datablock(s) I. DOI: 10.1107/S1600536812007350/hb6642Isup2.hkl

Supplementary material file. DOI: 10.1107/S1600536812007350/hb6642Isup3.cml

Additional supplementary materials:  crystallographic information; 3D view; checkCIF report

## Figures and Tables

**Table 1:** Hydrogen-bond geometry (Å, °)

*D*—H⋯*A*	*D*—H	H⋯*A*	*D*⋯*A*	*D*—H⋯*A*
C11—H11⋯O1^i^	0.95	2.54	3.4530 (15)	161

Symmetry code: (i) 
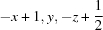
.

**Fig. 1.**
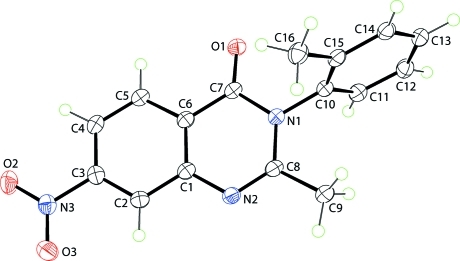
The molecular structure of (I) showing displacement ellipsoids at the 50% probability level.

**Fig. 2.**
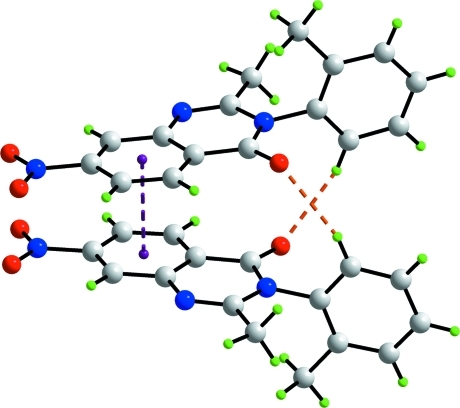
A view of the two-molecular aggregate in (I) sustained by C—H···O and π–π interactions shown as orange and purple dashed lines, respectively.

**Fig. 3.**
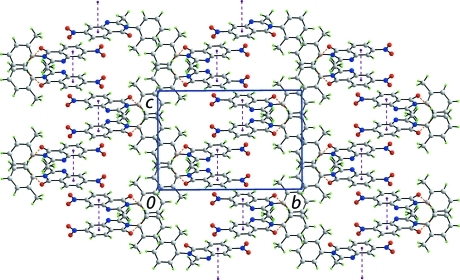
A view of the assembly of two-molecule aggregates in the bc plane in (I). The C—H···O and π–π interactions are shown as orange and purple dashed lines, respectively.

**Fig. 4.**
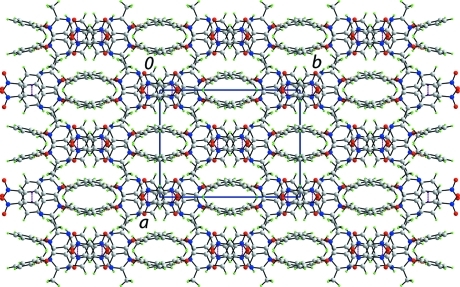
A view in projection down the a axis of the unit-cell contents of (I). The C—H···O and π–π interactions are shown as orange and purple dashed lines, respectively.

## supplementary crystallographic information

### Comment

The title methaqualone analogue, 2-methyl-7-nitro-3-*o*-tolylquinazolin-4(3*H*)-one (I), has been investigated previously for its anti-convulsant activity (El-Azab *et al.*, 2012) as quinazoline-4(3*H*)-one derivatives are known for their various biological activities (El-Azab *et al.*, 2011, 2010). Herein, the crystal and molecular structure of (I) is described. A related structure with a similar conformation has been reported recently (Stephenson *et al.*, 2011).

The ten atoms comprising the fused ring system in (I), Fig. 1, are almost co-planar with the r.m.s. deviation for the non-hydrogen atoms being 0.029 Å. The 2-tolyl group is approximately orthogonal to this plane, forming a dihedral angle of 85.20 (5) °. The nitro group is almost co-planar with the ring to which it is connected as seen in the value of the O2—N3—C3—C2 torsion angle of 179.07 (10)°.

The main feature of the crystal packing is the formation of two molecule aggregates sustained by C—H···O, Table 1, and π–π interactions, Fig. 2. The π–π interactions occur between (C1–C6) rings, *i.e*. the C_6_ group within the fused ring system, with a ring centroid-to-centroid distance of 3.4967 (6) Å [symmetry operation: 1 - *x*, *y*, 1/2 - *z*]. There are no specific intermolecular interactions between the two-molecule aggregates [generated by 2-fold symmetry]. They assemble into layers in the *bc* plane, Fig. 3, and these stack along the *a* axis, Fig. 4.

### Experimental

4-Nitroanthranilic acid (1.82 g, 10 mmol) was refluxed with acetic anhydride (20 ml) for 3 h. The reaction mixture was cooled, filtered, washed with petroleum ether, and dried to yield 2-methyl-7-nitro-4*H*-3,1-benzoxazin-4-one as an a solid compound. This was refluxed with *o*-toludine (1.18 g, 11 mmol) in pyridine (30 ml) for 8 h. The reaction mixture was cooled, the solvent was removed under reduced pressure and the residue was triturated with water and filtered. The solid obtained was dried, chromatographed with CHCl_3_ and recrystallized from its EtOH solution as colourless cubes.

### Refinement

Carbon-bound H-atoms were placed in calculated positions [C—H = 0.95 to 0.98 Å, *U*_iso_(H) = 1.2 to 1.5*U*_eq_(C)] and were included in the refinement in the riding model approximation.

### Crystal data

**Table:**

C_16_H_13_N_3_O_3_	*F*(000) = 1232
*M**_r_* = 295.29	*D*_x_ = 1.444 Mg m^−^^3^
Monoclinic, *C*2/*c*	Cu *K*α radiation, λ = 1.54184 Å
Hall symbol: -C 2yc	Cell parameters from 3546 reflections
*a* = 14.4614 (5) Å	θ = 3.9–76.4°
*b* = 16.5383 (4) Å	µ = 0.85 mm^−^^1^
*c* = 12.9968 (4) Å	*T* = 100 K
β = 119.072 (4)°	Cube, colourless
*V* = 2716.78 (14) Å^3^	0.25 × 0.25 × 0.25 mm
*Z* = 8	

### Data collection

**Table:**

Agilent SuperNova Dual diffractometer with an Atlas detector	2783 independent reflections
Radiation source: SuperNova (Cu) X-ray Source	2570 reflections with *I* > 2σ(*I*)
Mirror monochromator	*R*_int_ = 0.013
Detector resolution: 10.4041 pixels mm^-1^	θ_max_ = 76.6°, θ_min_ = 4.4°
ω scan	*h* = −18→17
Absorption correction: multi-scan (*CrysAlis PRO*; Agilent, 2011)	*k* = −20→18
*T*_min_ = 0.967, *T*_max_ = 0.998	*l* = −10→16
5450 measured reflections	

### Refinement

**Table:**

Refinement on *F*^2^	Primary atom site location: structure-invariant direct methods
Least-squares matrix: full	Secondary atom site location: difference Fourier map
*R*[*F*^2^ > 2σ(*F*^2^)] = 0.035	Hydrogen site location: inferred from neighbouring sites
*wR*(*F*^2^) = 0.099	H-atom parameters constrained
*S* = 1.02	*w* = 1/[σ^2^(*F*_o_^2^) + (0.0566*P*)^2^ + 1.6285*P*] where *P* = (*F*_o_^2^ + 2*F*_c_^2^)/3
2783 reflections	(Δ/σ)_max_ < 0.001
201 parameters	Δρ_max_ = 0.24 e Å^−^^3^
0 restraints	Δρ_min_ = −0.24 e Å^−^^3^

### Special details

**Table:**

Geometry. All e.s.d.'s (except the e.s.d. in the dihedral angle between two l.s. planes) are estimated using the full covariance matrix. The cell e.s.d.'s are taken into account individually in the estimation of e.s.d.'s in distances, angles and torsion angles; correlations between e.s.d.'s in cell parameters are only used when they are defined by crystal symmetry. An approximate (isotropic) treatment of cell e.s.d.'s is used for estimating e.s.d.'s involving l.s. planes.
Refinement. Refinement of *F*^2^ against ALL reflections. The weighted *R*-factor *wR* and goodness of fit *S* are based on *F*^2^, conventional *R*-factors *R* are based on *F*, with *F* set to zero for negative *F*^2^. The threshold expression of *F*^2^ > σ(*F*^2^) is used only for calculating *R*-factors(gt) *etc*. and is not relevant to the choice of reflections for refinement. *R*-factors based on *F*^2^ are statistically about twice as large as those based on *F*, and *R*- factors based on ALL data will be even larger.

### Fractional atomic coordinates and isotropic or equivalent isotropic displacement parameters (Å^2^)

**Table:**

	*x*	*y*	*z*	*U*_iso_*/*U*_eq_	
O1	0.59490 (6)	0.19268 (5)	0.43782 (7)	0.0250 (2)	
O2	0.65765 (7)	0.61062 (5)	0.45232 (9)	0.0338 (2)	
O3	0.48735 (7)	0.62439 (5)	0.36278 (8)	0.0330 (2)	
N1	0.41702 (7)	0.21605 (6)	0.35394 (8)	0.0201 (2)	
N2	0.34237 (7)	0.34733 (6)	0.30533 (8)	0.0217 (2)	
N3	0.56777 (8)	0.58294 (6)	0.40625 (9)	0.0247 (2)	
C1	0.44368 (9)	0.37812 (7)	0.35225 (9)	0.0196 (2)	
C2	0.45504 (9)	0.46277 (7)	0.35369 (9)	0.0208 (2)	
H2	0.3949	0.4972	0.3210	0.025*	
C3	0.55556 (9)	0.49398 (7)	0.40369 (10)	0.0214 (2)	
C4	0.64664 (9)	0.44647 (7)	0.45173 (10)	0.0225 (2)	
H4	0.7148	0.4707	0.4859	0.027*	
C5	0.63564 (9)	0.36354 (7)	0.44855 (9)	0.0215 (2)	
H5	0.6964	0.3298	0.4795	0.026*	
C6	0.53454 (9)	0.32924 (7)	0.39953 (9)	0.0195 (2)	
C7	0.52197 (8)	0.24124 (7)	0.39980 (9)	0.0200 (2)	
C8	0.33210 (8)	0.26981 (7)	0.30874 (9)	0.0211 (2)	
C9	0.22363 (9)	0.23499 (7)	0.26233 (11)	0.0273 (3)	
H9A	0.1715	0.2788	0.2356	0.041*	
H9B	0.2199	0.2044	0.3248	0.041*	
H9C	0.2084	0.1989	0.1962	0.041*	
C10	0.39793 (8)	0.13076 (7)	0.36267 (10)	0.0207 (2)	
C11	0.37329 (9)	0.08065 (7)	0.26703 (10)	0.0236 (2)	
H11	0.3732	0.1011	0.1987	0.028*	
C12	0.34882 (9)	0.00019 (7)	0.27286 (11)	0.0252 (3)	
H12	0.3291	−0.0345	0.2071	0.030*	
C13	0.35326 (9)	−0.02949 (7)	0.37522 (11)	0.0242 (3)	
H13	0.3371	−0.0846	0.3798	0.029*	
C14	0.38125 (9)	0.02132 (7)	0.47070 (10)	0.0232 (2)	
H14	0.3863	−0.0001	0.5411	0.028*	
C15	0.40209 (8)	0.10313 (7)	0.46597 (10)	0.0214 (2)	
C16	0.42659 (10)	0.15867 (8)	0.56764 (10)	0.0270 (3)	
H16A	0.4905	0.1899	0.5866	0.040*	
H16B	0.4381	0.1266	0.6363	0.040*	
H16C	0.3671	0.1957	0.5463	0.040*	

### Atomic displacement parameters (Å^2^)

**Table:**

	*U*^11^	*U*^22^	*U*^33^	*U*^12^	*U*^13^	*U*^23^
O1	0.0190 (4)	0.0239 (4)	0.0292 (4)	0.0027 (3)	0.0094 (3)	0.0001 (3)
O2	0.0311 (5)	0.0281 (5)	0.0405 (5)	−0.0096 (4)	0.0161 (4)	−0.0045 (4)
O3	0.0324 (5)	0.0247 (4)	0.0405 (5)	0.0026 (4)	0.0166 (4)	0.0039 (4)
N1	0.0172 (5)	0.0203 (5)	0.0208 (5)	−0.0004 (3)	0.0077 (4)	0.0000 (3)
N2	0.0180 (4)	0.0233 (5)	0.0217 (5)	0.0007 (4)	0.0080 (4)	0.0022 (4)
N3	0.0284 (5)	0.0241 (5)	0.0238 (5)	−0.0033 (4)	0.0143 (4)	−0.0008 (4)
C1	0.0190 (5)	0.0242 (5)	0.0158 (5)	0.0000 (4)	0.0087 (4)	0.0003 (4)
C2	0.0213 (5)	0.0237 (6)	0.0182 (5)	0.0017 (4)	0.0101 (4)	0.0015 (4)
C3	0.0265 (6)	0.0218 (5)	0.0183 (5)	−0.0021 (4)	0.0126 (4)	−0.0007 (4)
C4	0.0195 (5)	0.0275 (6)	0.0194 (5)	−0.0046 (4)	0.0088 (4)	−0.0023 (4)
C5	0.0185 (5)	0.0262 (6)	0.0187 (5)	0.0003 (4)	0.0082 (4)	−0.0005 (4)
C6	0.0194 (5)	0.0230 (5)	0.0160 (5)	−0.0002 (4)	0.0087 (4)	−0.0005 (4)
C7	0.0170 (5)	0.0245 (6)	0.0173 (5)	0.0003 (4)	0.0075 (4)	−0.0010 (4)
C8	0.0171 (5)	0.0251 (5)	0.0188 (5)	0.0004 (4)	0.0069 (4)	0.0014 (4)
C9	0.0175 (5)	0.0265 (6)	0.0326 (6)	−0.0001 (4)	0.0080 (5)	0.0038 (5)
C10	0.0161 (5)	0.0208 (5)	0.0226 (5)	−0.0002 (4)	0.0073 (4)	0.0006 (4)
C11	0.0216 (5)	0.0262 (6)	0.0217 (5)	−0.0001 (4)	0.0094 (4)	0.0001 (4)
C12	0.0223 (6)	0.0252 (6)	0.0260 (6)	−0.0021 (4)	0.0101 (5)	−0.0049 (4)
C13	0.0182 (5)	0.0221 (5)	0.0291 (6)	−0.0014 (4)	0.0090 (5)	0.0007 (4)
C14	0.0164 (5)	0.0269 (6)	0.0240 (6)	−0.0010 (4)	0.0081 (4)	0.0030 (4)
C15	0.0145 (5)	0.0252 (6)	0.0219 (5)	−0.0002 (4)	0.0068 (4)	−0.0010 (4)
C16	0.0270 (6)	0.0301 (6)	0.0227 (6)	−0.0035 (5)	0.0112 (5)	−0.0025 (5)

### Geometric parameters (Å, º)

**Table:**

O1—C7	1.2227 (14)	C8—C9	1.4955 (15)
O2—N3	1.2253 (13)	C9—H9A	0.9800
O3—N3	1.2261 (14)	C9—H9B	0.9800
N1—C8	1.3938 (14)	C9—H9C	0.9800
N1—C7	1.3976 (14)	C10—C11	1.3887 (16)
N1—C10	1.4522 (14)	C10—C15	1.3915 (16)
N2—C8	1.2939 (15)	C11—C12	1.3885 (16)
N2—C1	1.3818 (14)	C11—H11	0.9500
N3—C3	1.4803 (14)	C12—C13	1.3899 (17)
C1—C6	1.4046 (15)	C12—H12	0.9500
C1—C2	1.4086 (15)	C13—C14	1.3863 (17)
C2—C3	1.3724 (16)	C13—H13	0.9500
C2—H2	0.9500	C14—C15	1.3939 (16)
C3—C4	1.3939 (16)	C14—H14	0.9500
C4—C5	1.3790 (16)	C15—C16	1.5035 (15)
C4—H4	0.9500	C16—H16A	0.9800
C5—C6	1.4000 (15)	C16—H16B	0.9800
C5—H5	0.9500	C16—H16C	0.9800
C6—C7	1.4670 (15)		
			
C8—N1—C7	122.78 (9)	C8—C9—H9A	109.5
C8—N1—C10	119.37 (9)	C8—C9—H9B	109.5
C7—N1—C10	117.68 (9)	H9A—C9—H9B	109.5
C8—N2—C1	117.70 (9)	C8—C9—H9C	109.5
O2—N3—O3	124.04 (10)	H9A—C9—H9C	109.5
O2—N3—C3	117.96 (10)	H9B—C9—H9C	109.5
O3—N3—C3	117.99 (10)	C11—C10—C15	122.65 (11)
N2—C1—C6	123.15 (10)	C11—C10—N1	119.05 (10)
N2—C1—C2	117.64 (10)	C15—C10—N1	118.27 (10)
C6—C1—C2	119.20 (10)	C12—C11—C10	118.98 (11)
C3—C2—C1	118.02 (10)	C12—C11—H11	120.5
C3—C2—H2	121.0	C10—C11—H11	120.5
C1—C2—H2	121.0	C11—C12—C13	119.74 (11)
C2—C3—C4	123.58 (10)	C11—C12—H12	120.1
C2—C3—N3	118.14 (10)	C13—C12—H12	120.1
C4—C3—N3	118.28 (10)	C14—C13—C12	120.02 (11)
C5—C4—C3	118.49 (10)	C14—C13—H13	120.0
C5—C4—H4	120.8	C12—C13—H13	120.0
C3—C4—H4	120.8	C13—C14—C15	121.61 (11)
C4—C5—C6	119.76 (10)	C13—C14—H14	119.2
C4—C5—H5	120.1	C15—C14—H14	119.2
C6—C5—H5	120.1	C10—C15—C14	116.90 (10)
C5—C6—C1	120.93 (10)	C10—C15—C16	121.90 (10)
C5—C6—C7	120.27 (10)	C14—C15—C16	121.19 (10)
C1—C6—C7	118.77 (10)	C15—C16—H16A	109.5
O1—C7—N1	121.42 (10)	C15—C16—H16B	109.5
O1—C7—C6	124.68 (10)	H16A—C16—H16B	109.5
N1—C7—C6	113.88 (9)	C15—C16—H16C	109.5
N2—C8—N1	123.66 (10)	H16A—C16—H16C	109.5
N2—C8—C9	118.89 (10)	H16B—C16—H16C	109.5
N1—C8—C9	117.45 (10)		
			
C8—N2—C1—C6	2.02 (16)	C1—C6—C7—O1	179.56 (10)
C8—N2—C1—C2	−176.98 (10)	C5—C6—C7—N1	176.62 (9)
N2—C1—C2—C3	177.87 (10)	C1—C6—C7—N1	−1.68 (14)
C6—C1—C2—C3	−1.17 (15)	C1—N2—C8—N1	−1.83 (16)
C1—C2—C3—C4	0.77 (17)	C1—N2—C8—C9	177.94 (10)
C1—C2—C3—N3	−179.48 (9)	C7—N1—C8—N2	−0.21 (17)
O2—N3—C3—C2	179.07 (10)	C10—N1—C8—N2	174.96 (10)
O3—N3—C3—C2	−0.50 (15)	C7—N1—C8—C9	−179.98 (10)
O2—N3—C3—C4	−1.17 (15)	C10—N1—C8—C9	−4.82 (15)
O3—N3—C3—C4	179.26 (10)	C8—N1—C10—C11	88.12 (13)
C2—C3—C4—C5	0.36 (17)	C7—N1—C10—C11	−96.47 (12)
N3—C3—C4—C5	−179.39 (9)	C8—N1—C10—C15	−90.00 (12)
C3—C4—C5—C6	−1.07 (16)	C7—N1—C10—C15	85.41 (13)
C4—C5—C6—C1	0.66 (16)	C15—C10—C11—C12	1.87 (17)
C4—C5—C6—C7	−177.60 (10)	N1—C10—C11—C12	−176.16 (10)
N2—C1—C6—C5	−178.50 (10)	C10—C11—C12—C13	−2.42 (17)
C2—C1—C6—C5	0.48 (16)	C11—C12—C13—C14	0.49 (17)
N2—C1—C6—C7	−0.22 (15)	C12—C13—C14—C15	2.14 (17)
C2—C1—C6—C7	178.77 (9)	C11—C10—C15—C14	0.65 (16)
C8—N1—C7—O1	−179.25 (10)	N1—C10—C15—C14	178.70 (9)
C10—N1—C7—O1	5.51 (15)	C11—C10—C15—C16	−178.64 (10)
C8—N1—C7—C6	1.94 (14)	N1—C10—C15—C16	−0.59 (16)
C10—N1—C7—C6	−173.30 (9)	C13—C14—C15—C10	−2.66 (16)
C5—C6—C7—O1	−2.15 (17)	C13—C14—C15—C16	176.63 (10)

### Hydrogen-bond geometry (Å, º)

**Table:**

*D*—H···*A*	*D*—H	H···*A*	*D*···*A*	*D*—H···*A*
C11—H11···O1^i^	0.95	2.54	3.4530 (15)	161

Symmetry code: (i) −*x*+1, *y*, −*z*+1/2.
